# The double tragedy of agriculture vulnerability to climate variability in Africa: How vulnerable is smallholder agriculture to rainfall variability in Ghana?

**DOI:** 10.4102/jamba.v8i3.249

**Published:** 2016-04-19

**Authors:** Emmanuel K. Derbile, Dramani J.M. File, Alfred Dongzagla

**Affiliations:** 1 Department of Planning and Management, University for Development Studies, Ghana; 2 National Disaster Management Organization, Wa, Ghana

## Abstract

This article analysed vulnerability of smallholder agriculture to climate variability, particularly the alternating incidences of drought and heavy precipitation events in Ghana. Although there is an unmet need for understanding the linkages between climate change and livelihoods, the urgent need for climate change adaptation planning (CCAP) in response to climate change makes vulnerability assessment even more compelling in development research. The data for analysis were collected from two complementary studies. These included a regional survey in the Upper West Region and an in-depth study in three selected communities in the Sissala East District. The results showed that smallholder agriculture is significantly vulnerable to climate variability in the region and that three layers of vulnerability can be identified in a ladder of vulnerability. Firstly, farmers are confronted with the double tragedy of droughts and heavy precipitation events, which adversely affect both crops and livestock. Secondly, farmers have to decide on crops for adaptation, but each option – whether indigenous crops, new early-maturing crops or genetically modified crops – predisposes farmers to a different set of risks. Finally, the overall impact is a higher-level vulnerability, namely the risk of total livelihood failure and food insecurity. The article recommended CCAP and an endogenous development (ED) approach to addressing agriculture vulnerability to climate variability within the framework of decentralisation and local governance in Ghana.

## Introduction

Although the evidence on global climate change is overwhelming, there is still an unmet need to understand climate change and the associated vulnerabilities at local level for informing development planning, particularly in Africa. According to Giddens (2008), there are still competing debates about climate change. One position is that present-day global warming processes are caused by natural factors consistent with world climate history. The opposing view is that climate change is caused by human factors and the threats exaggerated if compared with other global problems such as poverty, Aids and the risk of nuclear weapons. This article is situated within the context that climate change is occurring and that it presents some challenges to development and human security. In particular, it analyses the vulnerability of agriculture, particularly crop production, to climate variability in the Upper West Region of Ghana and the policy implications for climate change adaptation planning (CCAP) under decentralisation in Ghana and Africa at large.

Africa is one of the most vulnerable continents to climate change in the world. Rising global temperatures threaten the hydrological cycle and may cause both dry and wet conditions. This will increase drought risks and floods globally and compromise access to quality and quantity of water for one-sixth of the world’s population, including Africa (WaterAid 2007). These changes in precipitation will cause severe water shortages across the world (Stern 2006; UNFCCC 2007). Severe drought conditions in the African Sahel are anticipated to continue whilst the eastern and western parts of Africa will be experiencing more precipitation. Precipitation in sub-Saharan Africa is showing decreasing trends with other extreme variations, which have dire consequences for agriculture and food security. These variations translate in shorter rainy seasons, uncertainty and unpredictable rainfall patterns. These increase the risk and frequency of droughts and other climatic hazards in the region (Gbetibouo 2009; Mengistu 2011). Thus, the situation regarding climate variability in Africa, particularly sub-Saharan Africa, reveals a mix of extremes in dry and wet conditions. A report on Tanzania’s climate change suggests that areas that experience two rainfall regimes will have more rainfall whilst areas with one rainfall regime will have less (Chauhan 2010).

The greatest challenge, deserving more attention in the climate change discourse, is the effects of climate change on livelihoods, particularly agriculture. According to the UNFCCC (2007), about 75–220 million people in Africa will be severely affected by drought by the year 2020. The repercussions of this on agriculture are severe as about 90% of agriculture in Africa, especially sub-Saharan Africa, is mainly rain-fed (Chauhan 2010; Nyong, Adesina & Elasha 2007; WaterAid 2007). For instance, double exposure to recurrent floods and droughts in Africa would adversely affect smallholder agriculture and food security (Armah *et al*. 2010; Chauhan 2010). In the West-African sub-region, agriculture, which employs about 290 million people (about 60% of the workforce) and accounts for 35% of the gross domestic product (GDP), is being endangered by climate change (Jalloh *et al*. 2013). According to Stern (2006), a 2 °C rise in temperature will likely result in exposing 1–4 billion people across the world, with the majority coming from Africa and other developing continents, to water shortage. For instance, it is estimated that extreme climatic events will reduce the net yield of maize in Tanzania by 33% (Chauhan 2010).

Agriculture vulnerability in Africa arises largely from dependence on rain-fed agriculture and thus susceptibility of production to changes in precipitation and low technological capacity for adaptation. For instance, in sub-Saharan Africa, the majority of the people are engaged in smallholder farming and livestock rearing as primary livelihoods. However, access to tractors and other modern implements and inputs such as quality seeds, fertilisers and pesticides are beyond the reach of most poor farmers in the sub-region (Care International 2011). Low capacities in terms of income and technology arise from high poverty levels in many African countries (Care International 2011; Jalloh *et al*. 2013).

### Vulnerability context: Study area, climate and agriculture

**FIGURE 1:**
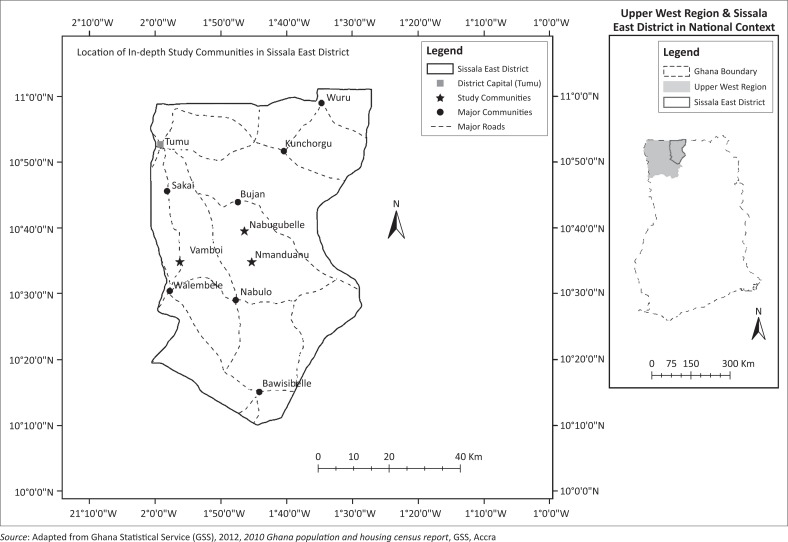
Map showing study area in national context.

This article addresses the subject of agriculture vulnerability in the Upper West Region of Ghana in the context of climate and agriculture (Figure 1). The region falls within the Guinea Savannah vegetation and tropical continental climatic belts.

The region is characterised by a single maximum rainfall regime, usually from May to September, but the patterns have become variable and unpredictable. The climate is pointing to a drying trend in which both extreme dry and wet events occur, as is the case for the sub-region and continent as a whole. The rainfall pattern in Ghana has become irregular and difficult to predict because of climate variability (Kankam-Yeboah, Amisigo & Obuobi 2010).

For instance, in Sissala East District, the total number of days of rain ranged between 70 and 80 days in the 1990s, but only 51 days were recorded for 2009. Similarly, the mean annual rainfall was 121 mm in 1999 as compared to 104 mm in 2009 (Sissala East District Assembly 2010, 2013). There is an indication that the number of days of rain as well as the mean annual rainfall are decreasing in the district. This has implications for food security in the district. The rainy season starts late and stops abruptly or early. This results in dry conditions with negative consequences for farm crops and livestock. In some instances, there have been extremely heavy rainfalls, particularly during the peak of the rainy season, resulting in floods (Kankam-Yeboah *et al*. 2010).

The most daunting challenge of climate variability is its effect on agriculture. In Ghana, particularly, northern Ghana, rain-fed agriculture is the primary source of livelihood for a majority of the population. Hence, extreme climatic events such as droughts, heavy rainfall and floods tend to have a widespread damaging effect on agriculture, disrupting the livelihoods of the majority of households in northern Ghana. For instance, heavy precipitation events combine with the annual spillage of the Bagre and Kompienga Dams in neighbouring Burkina Faso to affect hundreds of acres of farmlands, especially in the Upper East and northern Regions (Kankam-Yeboah *et al*. 2010). The floods that occurred in northern Ghana in 2007 affected about 332 600 people and caused the death of 56 people in the Upper East, Upper West and northern regions and parts of the western Region (Kankam-Yeboah *et al*. 2010; Republic of Ghana 2013). Thus, climate variability coupled with the inability of farmers to predict rainfall patterns increase farmers’ exposure to climatic risk and hazards and result in crop failure and loss of livestock (Andreini *et al*. 2000; Laube, Leemhuis & Amisigo 2008:2).

### Vulnerability: Theoretical and conceptual framework

This article draws on ‘vulnerability’ as an integrated concept which provides an overarching theoretical and conceptual framing for analysing vulnerability of agriculture to climate variability. The United Nations International Strategy for Disaster Reduction (UNISDR) underpins the integrated nature of vulnerability. It defines vulnerability as the characteristics and circumstances of a community, system or asset that make it susceptible to the damaging effects of a hazard. Further to this, it explains that vulnerability embodies many factors, including physical, social, economic and environmental factors (UNISDR 2004:12). Vulnerability is conceptualised in the context of susceptibility of agriculture to climate change and climate variability. The Intergovernmental Panel on Climate Change (IPCC), in its Second Assessment Report, defines vulnerability as ‘the extent to which climate change may damage or harm a system’ (Olmos 2001:2). In another instance, the IPCC defined vulnerability as the degree to which a system is susceptible to or unable to cope with the adverse effects of climate change, including climate variability as a result of adaptive capacity (Parry *et al*. 2007:783). Similarly, the International Federation of Red Cross and Red Crescent Societies (IFRC) define vulnerability as the diminished capacity of an individual or group to anticipate, cope with, resist and recover from the damaging effect of a natural or man-made hazard (IFRC 2009). Vulnerability is most often linked to poverty, but it can also emerge when people are isolated, insecure and defenceless in the event of risk, shock or stress (IFRC 2009). According to Cannon, Twigg and Rowell (2003), vulnerability involves much more than people being injured or killed in the event of a hazard; it also includes the impacts of hazards on the livelihood of people.

An important dimension of vulnerability is that of differentiation. A common theme in climate change literature is that countries, regions, economic sectors and social groups differ in their degree of vulnerability to climate change (IFRC 2009; Olmos 2001). This is partly because changes in temperature and rainfall will occur unevenly and that climate change impacts will be unevenly distributed around the globe (Olmos 2001). It is also because resources and wealth are distributed unequally (Olmos 2001). Olmos (2001) argues that the study of adaptation to climate change should begin with the study of social and economic vulnerability. According to the IFRC (2009), two questions are required for determining vulnerability: firstly, what threat or hazard are people exposed to; and secondly, what makes people vulnerable to these threats or hazards?

The need to analyse and prepare for addressing people’s vulnerability to disasters is well rooted in the DFID’s Sustainable Livelihood Framework (DFID 1999). The Sustainable Livelihood Framework presents the main factors that affect people’s livelihoods, and typical relationships between them. Relationships within the framework are highly complex (DFID 1999). Firstly, livelihood assets are both destroyed and created because of the trends, shocks and seasonality of the vulnerability context. Secondly, the institutions and policies of transforming structures and processes have a profound influence on access to assets. They create assets, determine access to assets and influence rates of asset accumulation. However, this is not a simple one-way relationship. Individuals and groups themselves influence transforming structures and processes. Generally speaking, the greater people’s asset endowment, the more influence they can exert. Hence, one way to achieve empowerment may be to support people to build up their assets. Thirdly, those with more assets tend to have a greater range of options and an ability to switch between multiple strategies to secure their livelihoods.

The analytical framework of this article is informed by the conception of vulnerability as a dual structure (see Birkman 2006; Bohle 2001; Chambers 2006; Van Dillen 2004). Vulnerability in this context is described as comprising two sides. The first is an external side which comprises the exposure to risks, shocks and stress which an individual or household experiences. The second is an internal side, a state of defencelessness arising from a low capacity to cope and/or adapt without damaging loss (Chambers 2006:33; also see Bohle 2001; Van Dillen 2004). This dual structure of vulnerability was adapted to analyse the vulnerability of agriculture to climate variability in north-western Ghana. Thus, three interrelated conceptual and methodological questions provided guidance for analysis:

- What risk factors do farmers encounter in smallholder agriculture as a result of climate change and climate variability?
- How do these risk factors affect smallholder agriculture production?
- How can vulnerability to climate variability in smallholder agriculture be differentiated?

## Methodology

This article draws on a mixed research design comprising a qualitative study and a quantitative study. These two approaches complemented each other.

A qualitative study was conducted in three randomly selected communities, namely Vamboi, Nabugbelle and Nmanduanu in the Sissala East District (Figure 1). Firstly, in-depth interviews were conducted amongst 150 households with the aid of an interview guide. Systematic random sampling was applied in sampling households. At the household level, stratified random sampling was applied for interviewing respondents. Thus, the interviews covered 80 male heads of households and 37 female spouses, and 27 female heads of households. The second level of in-depth interviews was conducted amongst 28 key informants (KIs). These KIs included opinion leaders, namely chiefs, elders, earth priests ( *jantina*), community chief farmers, and women leaders such as *magazia*. The others included leaders of women groups, and male and female youth leaders. Household heads and KIs were targeted for their experience and knowledge regarding farming. The data collected centred on their experiences and observations of climate variability and its effect on agriculture.

In addition to the interviews, focus group discussions (FGDs) were conducted amongst three categories of discussants in each community. These included the chiefs and council of elders, adult male farmers and adult female farmers. The others included male youth and female youth farmers. Overall, 15 FGDs were conducted with the number of discussants ranging between 7 and 12 per session. An FGD guide was used for guiding the discussions. Overall, the FGDs enabled in-depth discussions and analysis of local perspectives on the causes of climate variability at all study sites.

The article also draws on a regional survey conducted in the Upper West Region (Figure 1). The study covered 540 randomly sampled households in 18 communities across six districts in the region. The study districts included Jirapa, Lambussie- Karni, Wa West, Wa East, Sissala West and Sissala East. The data were analysed using computer-based software for statistical analysis, SPSS.

## Results

The results are presented in two parts. The results on drought risk in food crop farming are analysed and presented. This is followed by an analysis of risks associated with heavy precipitation and floods in food crop farming.

### Drought risk in food crop farming

The results show significant exposure of food crop farming to drought risk. Results from the in-depth study in the Sissala District show that farmers understand drought as a period (short or long) of lack or very little rains that negatively affect the growth and yield of crops. This means both the absence of rainfall for a couple of days and for two to three weeks may be considered a period of drought as it affects the growth of food crops and production of livestock. Farmers reveal that in extreme cases, prolonged droughts may occur for a period of one month or even more, and that such droughts can be expected virtually every production season in recent times. According to farmers, prolonged droughts of longer than two weeks can occur two to three times within the same year.

From a historical perspective, farmers recalled the 1983 drought, a severe drought that had a devastating effect on agriculture and the natural vegetation experienced in the country and across many neighbouring West African countries. They recalled experiences of total crop failure, disruption of livelihoods, food insecurity crisis and survival on international food aid. Farmers asserted that they have not experienced a drought of such magnitude, only recurring droughts that adversely affect agriculture and livelihoods.

**FIGURE 2:**
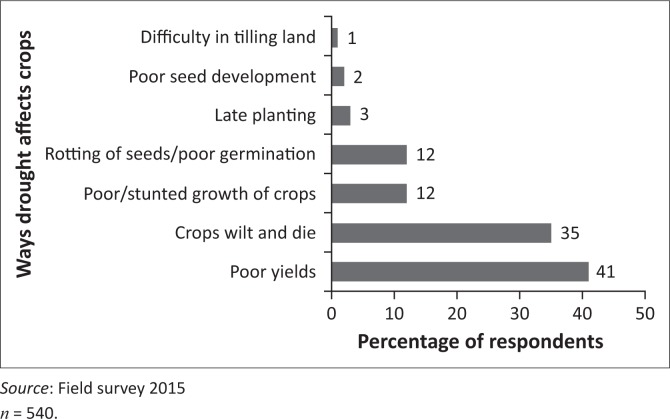
Effects of drought on food crop farming.

One of the key findings is that droughts hamper food crop farming in multiple ways. The survey identified seven specific effects of drought on food crop farming (Figure 2). The question posed to respondents was open and required respondents to identify the effects of drought from their experiences.

About 41% of respondents reported that droughts led to poor yields and another 35% reported that droughts caused crops to wilt and die. In addition, 12% of farmers attributed poor germination to drought and another 12% attributed poor growth to drought (Figure 2). In the survey, 100% of respondents reported experiencing annual food shortages arising from climate variability. This is because rain-fed agriculture is a primary source of food supply for household consumption.

Results from the qualitative study provide deeper insights into drought risks and food crop farming. Based on the results, we identified three periods of drought risk in the main production season. These include early droughts, mid-season droughts and late droughts.

Early droughts are associated with the planting season (June/July). These droughts usually occur before and after planting and arise from late onset of the rains. They generally affect planting and germination. Early droughts affect the production of beans (*bondaa*) greatly. The flowers fall prematurely, which affects pollination. They shed leaves prematurely and crops wilt and dry up at the time of developing fingers known in the local language as *kachu naniee*.

Mid-season droughts, comprising early mid-season and late mid-season droughts, occur in the middle of the raining season, in July and August. These droughts arise from extreme rainfall variability during the production season. When the droughts occur in the early mid-season, they hamper early weeding and application of fertiliser. When they occur in the late mid-season, they hamper late weeding, tussling of cereal crops, cob development and seed development. They also hamper the application of fertiliser, particularly sulphate of ammonia, which is meant for enhanced seed development.

Crop-specific analysis of the effects of drought provides further insights. For instance, mid-season drought leads to poor cob and seed formation in maize. Cobs may develop few seeds dotted on the cob, known in the local language as *korimi kanpolung*. Similarly, seed development of rice is compromised, as most do not develop seeds as a result of inadequate moisture. Legumes such as groundnuts and bambara beans and root and tuber crops such as yam and potatoes are unable to develop nuts and tubers, respectively. Groundnuts and bambara beans develop shells with no seeds, premature seeds or tiny seeds. In cases where seed development is poor, they cannot be consumed nor used as seed. In the case of yam and potatoes, tuber and root development, respectively, are poor. Roots and tubers are usually very tiny or lean. Farmers observed that cassava is drought resilient and is able to produce bigger and long roots under dry conditions.

Late droughts occur towards the end of the production season (September). Late-season droughts are very common and arise from an early or abrupt cessation in rainfall or very limited rainfall. They also hamper maturation and seeding. Tuber and root development is also truncated. Harvesting of crops such as groundnuts, bambara beans and sweet potatoes is hampered because the ground lacks the requisite moisture for uprooting.

### Heavy precipitation, floods and food crop farming

Based on the results, there are two forms of food crop farming risks related to heavy precipitation. The first is flash floods arising from heavy precipitation. This is the commonest form of flood, which occurs annually and may occur several times during the rainy season. The second is heavy floods arising from either prolonged heavy rainfall or high volumes of water originating from higher lands in Burkina Faso. This type of floods does not occur frequently. On average, heavy floods have occurred every 5–10 years over the past five decades. Farmers referred to the 2007/2008 floods that inundated most of the low-lying areas across the three regions in northern Ghana.

**FIGURE 3:**
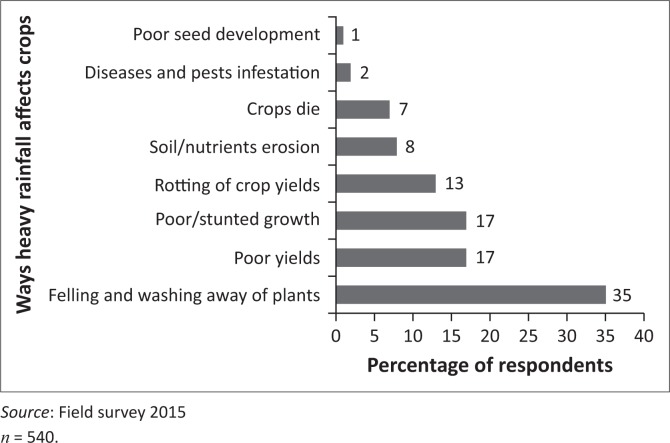
Effects of floods on food crop farming.

Both flash floods and heavy floods adversely affect food crop farming in different ways. Flash floods affect food crop production more frequently but the adverse effect is often far smaller than the effects of heavy floods arising from a long period of heavy rainfall. The survey identified eight adverse effects of floods on food crop farming (Figure 3).

Thirty-five per cent of respondents reported the felling and washing away of plants as an effect of floods on food crop farming. The other effects and corresponding percentages of respondents include poor yields (17%), stunted growth (17%), rotting of grains (13%) and soil erosion (8%).

The qualitative data provide some in-depth insight and/or specifics on the effects of heavy precipitation and floods on food crop farming. These can be summarised as follows:

- Inundation of farms. The first consequence of heavy precipitation and flash floods is that farms, especially those on low-lying lands, become inundated with water.
- Wilting and dying of crops. One of the effects of heavy rains and floods is that crops such as maize, millet, sorghum, guinea corn, cowpea, beans, groundnuts and bambara beans that are not water-loving usually shed leaves prematurely, wilt and finally die off.
- Poor seed and tuber development. The groundnut seeds shrink inside the shells and the shells change colour.
- Diseases affecting the crops. Millet, sorghum and guinea corn develop black pods (*javuuno*), which adversely affects seed development.
- Susceptibility of seeds, roots and tubers to rot. Early maize (*kuomifian*), beans (*bonda*) and groundnuts easily become rotten with heavy precipitation. Seeds begin to germinate as a result of incessant rainfall and heavy precipitation. Similarly, roots of cassava easily become rotten in waterlogged areas or under incessant heavy precipitation. Incessant heavy precipitation leads to lack of sunshine for drying grains and so they become moist and mouldy and rot in the process. Farmers recall experiences in years past in which they resorted to smoking maize as a mechanism for drying during heavy and incessant rains.
- The nuts of groundnuts begin to germinate inside the shells prior to harvest so that they cannot be used as seed. The demand for such groundnuts is compromised, as market prices are extremely low.
- Heavy precipitation may have significant effects on yam if the mounds are small. Yam plants that experience inundation and/or water logging develop smaller sized tubers. Farmers described these as ‘kwashiorkor-like’ tubers; the tubers develop big heads with very rough and thorny bodies with pointed ends. Such tubers are difficult to peel and they easily become rotten. However, farmers stressed that if the mounds are high and big, yams will develop big tubers in waterlogged areas. Similarly, potatoes and *yiiria* perform well under heavy precipitation. This is because they start to develop roots or tubers in September, when the rains are winding up. The tubers become fully matured after October.

#### Climate variability and threat to indigenous crop varieties

**FIGURE 4:**
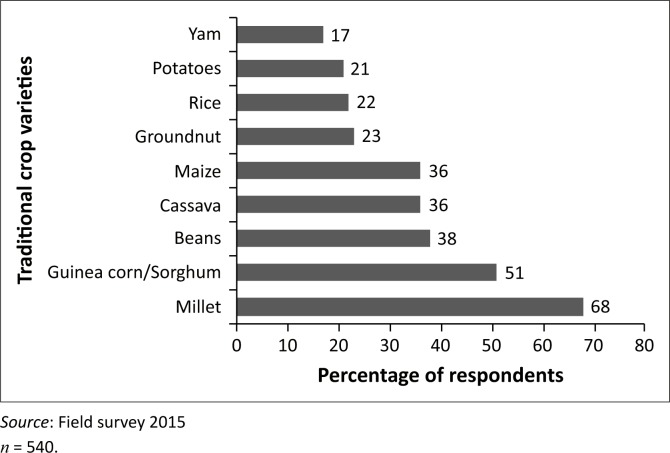
Indigenous crops some farmers have stopped cultivating.

One of the key findings from the study is that farmers are gradually abandoning the cultivation of some indigenous crop varieties because they are not adaptable to climate variability. The results from the survey identified ten such indigenous crop varieties (Figure 4).

About 68% of farmers reported that they had stopped cultivating the indigenous millet variety whilst almost 50% had stopped cultivating the indigenous guinea corn variety. Furthermore, between 17% and 38% of farmers have dropped various indigenous crop varieties from the portfolio of crops they plant.

Results from FGDs and in-depth interviews show that the production of traditional crop varieties of millet, guinea corn, sorghum, *mempeh* and *chati* have declined because of their limitations in adapting to climate variability. For instance, pollination of these crops is easily disrupted by heavy precipitation during tussling. This often results in poor seeding. Very often, such grains do not meet the requirements for use as seed in the next production season. They often develop black pods called *javuuno* in the Sissala area. A farmer summarised the problem of climate variability and the loss of indigenous seed varieties during an FGD session in Vamboi as follows:

‘These days, the rainfall pattern is highly uncertain and unpredictable and this has made us to lose our indigenous crops. We now cultivate modern and early maturing crop varieties but these are also more vulnerable to weeds, pests and disease. They are equally affected by rainfall variability. Rainfall variability also causes poor seed development in new crops and this adversely affects the marketing and market value of grains. We are always in a dilemma. Even these new crop varieties cannot do well under heavy rains and inadequate rainfall … so what do we do?’ (Farmer, male, 55 years)

### Vulnerability of agriculture to climate variability

The results show that agriculture is highly susceptible to climate variability in the study area, as is the case in most parts of Africa. Agriculture, particularly food crop farming but also poultry and livestock, is highly compromised by climate variability – a situation that threatens household food security.

**FIGURE 5:**
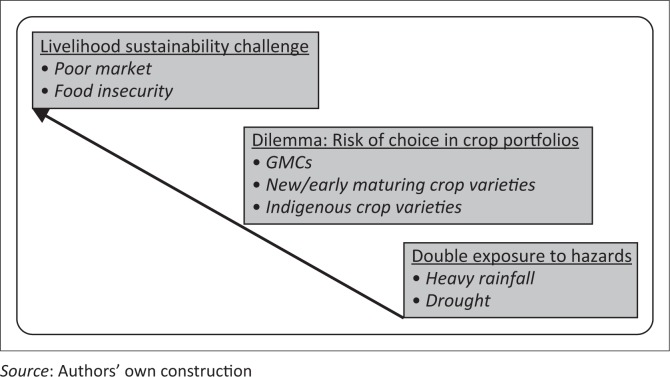
Ladder of agriculture vulnerability to climate variability.

Drawing inspiration from the principle of a ‘climate learning ladder’ (Tabara 2011), we analyse the vulnerability of agriculture to climate variability according to a ‘ladder of agriculture vulnerability’ to climate variability. Whilst Tabara (2011) applied the principle as an integrated and innovative assessment tool for appraising climate change and concepts for addressing the climate change challenge, we apply the concept to analysing vulnerability. Our ladder of vulnerability presents three interrelated layers of climate-related vulnerability in agriculture production (Figure 5).

In our ladder of vulnerability (Figure 5), the first level of vulnerable farmers encounter is the double exposure to droughts and heavy precipitation events and the double impacts on food crop, poultry and livestock production.^1^ The findings show that the recurrence of droughts and floods within the same farming season usually result in crop failure amongst many farmers. Upland farm crops are affected by droughts the most whilst lowland farm crops are affected by heavy precipitation and floods the most. This ultimately leads to poor yields and food insecurity in northern Ghana in particular. In the case of drought, farmer experiences show that most of the harm is caused by early and mid-season droughts because these are critical times of plant growth. The ultimate price of exposure to floods is very often total crop failure. Thus, we agree with Akudugu, Dittoh and Mahama (2012) that farmers in northern Ghana are usually hit by the double tragedy of floods and droughts in the same year. However, the double tragedy affects both sides of the vulnerability equation. On the one hand, farmers are exposed to the double hazards of drought and heavy precipitation events. On the other hand, crop, poultry and livestock production are compromised.

Although poultry and livestock rearing are very important livelihoods for many households in north-eastern Ghana, diseases undermine production (Derbile 2010) and some of these diseases are caused by extreme climatic events. Wilhite, Svoboda and Hayes (2007) note that high livestock mortality and reduced crop yields are direct impacts of drought. According to Mohammed, Derbile and Mordzeh-Ekpampo (2013), changes in weather patterns create conditions for emergence of new pests and diseases, which affect quality and quantity of yields of crops and livestock. In 2007, most of the landscape, especially low-lying farms in northern Ghana, was inundated by floods. The floods affected 70, 500 hectares and resulted in an estimated loss of 144 000 metric tonnes of food crops, namely maize, sorghum, millet, ground nuts, yam, cassava and rice (Armah *et al*. 2010). Furthermore, an estimated 50 000 vulnerable people experienced food insecurity and risked malnutrition. Armah *et al*. (2010) provide a vivid description of the effects of floods that reflect our findings:

The main consequence of flooding is the destruction of food crops on farms and seeds stores; eventually culminating in a decline in food production. A decline in food production can lead to starvation which may in some cases last for several months after each episode of floods leading to starvation. The reduction in food production also means loss of income for many in these communities which further reduce their ability to purchase food and thereby increasing the problem of food shortages and starvation within households. (p. 123)

The second layer of vulnerability is the dilemma farmers face in deciding on choice of crops for farming. Farmers have to consider three interrelated questions. The first is whether to maintain the production of indigenous crop varieties or not. The second is whether to adopt new crop varieties or not. The third is whether to adopt genetically modified crops (GMCs) or not. Farmers face a dilemma because each of these choices presents both opportunities and their own set of risks.

One of the key findings of this study is that farmers are abandoning the production of some indigenous crop varieties such as millet, maize, beans and cassava because of their limitations in adapting to climate variability. Farmers maintain mixed portfolios of crops in northern Ghana based on their understanding of their strengths and weaknesses for climate change adaptation. For instance, whilst some farmers still plant selected indigenous crop varies for adaptation to drought (Derbile 2013), others have adopted new, early-maturing crop varieties for same (Derbile 2010). Either choice presents both opportunities and risks. Firstly, although some indigenous crop varieties are drought resilient (Derbile 2013), extreme drought and heavy precipitation events adversely affect yields. However, indigenous crops are better suited to least external input agriculture akin to the poor resource production systems of smallholder farmers in Africa (Derbile, Jarawura & Dombo 2016). According to Nyantakyi-Frimpong (2013), the majority of farmers in northern Ghana use local crop varieties that require no chemical fertiliser application; farmers also believe local crops taste better. Derbile (2010) highlights similar farmer perspectives in north-eastern Ghana: that indigenous crop varieties are more suited to preparing traditional meals, and that they taste better and are most appropriate for traditional worship, sacrifices and funeral rites than new crop varieties.

The alternative is the adoption of new, early-maturing crop varieties; however, this has resource implications which can potentially alienate poor farmers and render them even more vulnerable. Most new crops, particularly maize and beans, which are adaptable to a shorter rainfall season, require the application of fertiliser and pesticides to guarantee a good harvest. However, most farmers cannot afford this added economic burden.

The primary goal of every smallholder farmer is to produce sufficient food for household consumption. However, this is often an unattainable goal for most farmers in northern Ghana because of the impact of climate variability. The third level of vulnerability addresses this challenge of livelihood sustainability in agriculture. Ultimately, climate variability adversely affects food crop production in two ways: it leads to low production, which translates into limited access, both physical and economic, to food. The exposure of agriculture to drought and heavy precipitation threaten food security and exacerbate poverty amongst smallholder farmers in north-western Ghana. This corroborates the assertion of Nelson and Agbey (2005) that climate variability increases poverty levels amongst subsistence farmers in rural Ghana. For instance, delay in the onset of rains is responsible for low yields of many major food crops in northern Ghana (Amikuzuno & Donkoh 2012). Hence, many households in northern Ghana experience food insecurity because of climate change (Akudugu *et al*. 2012).

Another critical effect of climate variability is that low production often leads to shortfalls in supply, culminating in price hikes in the foodstuff market. According to Mohammed *et al*. (2013), poor yields arising from climate variability affect availability and prices of food in northern Ghana. Thus, farmers are exposed to the risks of market or commodity price fluctuations and hence suffer a double commodity pricing system, especially from May to July. Devereux (2007) emphasises that droughts and floods have a strong negative effect on commodity markets. This is because weather shocks result in reduced harvests and cause food availability decline. As the demand for food is highly price-inelastic (food being a necessity), a relatively small shortfall in market supplies can cause a major increase in food prices. According to Derbile (2010), prices of farm produce, particularly sorghum and groundnuts, often increase as a result of low production and supply resulting from poor rains in north-eastern Ghana. These findings corroborate assertions of Kankam-Yeboah *et al*. (2010), Glantz, Gommes and Ramasamy (2009) and Boko *et al*. (2007) that climate change will affect the prices of cereals. Devereux (2007) sums up the dynamics in food prices in Africa in reference to the Malawian experience as follows:

Every year, food prices follow a predictable seasonal pattern: starting low after the main annual harvest in April–May, rising steadily through the calendar year and peaking during the ‘hungry season’ months of January–March. In 2001/2002, retail prices of maize and cassava started rising rapidly soon after the poor harvest and reached unprecedented heights in January 2002, more than 300% above the average post-harvest prices. (p. 51)

## Conclusion and recommendations for planning

The article concludes that smallholder agriculture is significantly vulnerable to climate variability in north-western Ghana. The article highlights three layers of vulnerability. Vulnerability begins with the double tragedy of farmer exposure to both droughts and heavy precipitation events. The next stage of vulnerability is associated with farmers’ decisions regarding the choices of crops for adaptation because each option – whether indigenous crops, new early-maturing crops or GMCs – predisposes farmers to different sets of risks. The overall impact of these levels of vulnerability is a higher-level vulnerability, namely risk of total livelihood failure and food insecurity amongst smallholder farmers.

The article recommends CCAP within the framework of district development planning (DDP) and local governance for sustaining climate change response for addressing vulnerability to climate variability in Ghana and Africa at large. The policy thrust should be to build capacity for adaptation to drought and heavy precipitation events. The implementation strategy should draw on an endogenous development (ED) approach, maximising the utilisation of local resources and existing local knowledge systems and practices and selectively borrowing knowledge, innovations and resources from external sources for complementing local initiatives in the development process.

## Footnotes

1 In terms of data, the paper focused more on the impact of climate variability on food crop production and less on poultry and livestock production.
